# Poisonous Bites

**Published:** 1859-10

**Authors:** 


### POISONOUS BITES.

DURING the increased travel of summer the bites from insects and reptiles of various kinds are of frequent occurrence. Persons of healthful blood are bitten with impunity sometimes, while those in feeble health suffer distressing, and sometimes fatal consequences.

Almost all poisonous bites arise from the acidity of the virus, it then follows that an alkali is the best antidote, because an alkali and an acid are as much opposed to each other as light and darkness, as sweet and sour. And as expedition is sometimes the life of a man, it is of considerable practical importance to know what is the most universally available remedy. A handful of the fresh ashes of wood is the most generally accessible, pour on enough water, hot is best, to cover it, stir it quickly, and either apply the fluid part, that is the ley, with a rag or sponge, or have less water, and apply a poultice made of simple water and fresh wood ashes. Renew the poultice every half hour until the hurting is entirely removed. As to minor insects, the relief is almost instantaneous. The next most convenient remedy is common spirits of hartshorn, a small vial of which should be in every family, and in every traveler's trunk or carpet bag, in summer time at least. Saleratus dampened and applied to the wound or stung place, is not as powerful as hartshorn. It failed recently to cure the sting of a bee, the gentleman dying in convulsions within an hour after he was stung; this arose from some peculiarity of constitution, an " Idiosyncrasy" as physicians term it.

